# Extracellular Matrix Metalloproteinase Inducer EMMPRIN (CD147) in Cardiovascular Disease

**DOI:** 10.3390/ijms19020507

**Published:** 2018-02-08

**Authors:** Saskia N. I. von Ungern-Sternberg, Alma Zernecke, Peter Seizer

**Affiliations:** 1 Medizinische Klinik III, Kardiologie und Kreislauferkrankungen, Eberhard Karls-Universität Tübingen, 72076 Tübingen, Germany; Saskia.Ungern-Sternberg@med.uni-tuebingen.de; 2 Institut für Experimentelle Biomedizin, Universitätsklinikum Würzburg, 97080 Würzburg, Germany

**Keywords:** cardiovascular disease, immunoglobulin superfamily, inflammation, platelets, monocyte-platelet aggregates

## Abstract

The receptor EMMPRIN is involved in the development and progression of cardiovascular diseases and in the pathogenesis of myocardial infarction. There are several binding partners of EMMPRIN mediating the effects of EMMPRIN in cardiovascular diseases. EMMPRIN interaction with most binding partners leads to disease progression by mediating cytokine or chemokine release, the activation of platelets and monocytes, as well as the formation of monocyte-platelet aggregates (MPAs). EMMPRIN is also involved in atherosclerosis by mediating the infiltration of pro-inflammatory cells. There is also evidence that EMMPRIN controls energy metabolism of cells and that EMMPRIN binding partners modulate intracellular glycosylation and trafficking of EMMPRIN towards the cell membrane. In this review, we systematically discuss these multifaceted roles of EMMPRIN and its interaction partners, such as Cyclophilins, in cardiovascular disease.

## 1. Introduction

The cluster of differentiation 147 (CD147) is a member of the immunoglobulin superfamily [1] and was first described in 1982 in a fibroblasts-tumor cell co-culture assay as a stimulator of matrix metalloproteinase (MMP)-1 production in fibroblasts [2]. In the years between 1982 and 1995, EMMPRIN was subsequently identified in different species and tissues and referred to by several names, i.e., ox-47 in rats [3], gp42 [4], basigin (basic immunoglobulin superfamily) [1], tumor cell collagenase-stimulatory factor (TCSF) in mice [5], HT7 [6] and Neurothelin [7] in chickens, and M6 [8] in humans. In 1995, Biswas et al. [9] renamed TCSF to EMMPRIN (Extracellular Matrix Metalloproteinase Inducer) to indicate its role in MMP induction. On activated platelets, EMMPRIN acts as an adhesion receptor [10]. EMMPRIN is upregulated on monocytes during differentiation into macrophages, and an enhanced expression of EMMPRIN has been described in human atherosclerotic plaques, especially in macrophage-rich regions [11].

In this review article, we systematically discuss the role of EMMPRIN and its interaction partners in cardiovascular diseases.

## 2. Structure and Cellular Location of EMMPRIN

Structurally, human EMMPRIN consists of 269 amino acids [12], comprising a signal peptide (21 amino acids), an extracellular domain (185 amino acids), a transmembrane domain (24 amino acids) and a cytoplasmic tail (39 amino acids) [9]. The extracellular part consists of two extracellular domains, an IgC2-type (EC1) domain at the N-terminal part of the extracellular domain and an IgI-type (EC2) domain at the C-terminal domain of the extracellular domain [13].

EMMPRIN can be glycosylated at three (N)-glycosylation sites, and distinguished according to its glycosylation state into a lowly and a highly glycosylated form [14]. (*N*)-glycosylation site Asn^44^ is located in the EC1 domain, and Asn^152^ and Asn^186^ are within the EC2 domain [1]. The lowly glycosylated form is about 32 kDa, the highly glycosylated form is between 45–65 kDa. Unglycosylated EMMPRIN has a size of 27 kDa [14]. Most common is the highly glycosylated form [15].

There are strong homologies between Ig domains of EMMPRIN and IgVκ and the β-chain of major histocompatibility complex (MHC) class II [1]. In addition, there is a high conservation of the transmembrane part across different species, suggesting an important functional role of the transmembrane part [8].

Intracellularly, EMMPRIN is located in the open canalicular system (OCS) and in α-granules of platelets. Platelet surface expression of EMMPRIN is upregulated after platelet activation with platelet agonists, e.g., thrombin or adenosine diphosphate [16]. Besides the membrane-bound form of EMMPRIN acting as a cell surface receptor, there is also a soluble form. Soluble EMMPRIN is either transported to the cell membrane via vesicular transport and then secreted into the extracellular space, or cleaved from the cell surface by proteolytic cleavage, e.g., by MT1-MMP [5,17].

## 3. EMMPRIN Binding Partners

EMMPRIN serves as a binding partner of several receptors and ligands. Known EMMPRIN ligands include Cyclophilin A (CyPA) [18], CyPB [19], S100A9 [20], monocarboxylate transporter (MCT)-1 and MCT4 [21], glycoprotein VI (GPVI) [10], CD98 [22], CD44 [23], CD43 [24], E-Selectin [25], CyP60 [26], Apolipoprotein D (ApoD) [27], Caveolin-1 [28], Annexin-2 [29] and NOD2 [30], syndecean-1 [31], as well as the integrin α^3^β_1_ and α^6^β_1_ [32].

In addition, EMMPRIN can form dimers with other EMMPRIN receptors, mainly via the EC1 domain on the cell membrane in a cis-dependent manner [33]. EMMPRIN dimer formation was reported to occur across platelets and monocytes as well as EMMPRIN-CHO cells and recombinant EMMPRIN-Fc under shear stress [10,34]. In the following, we will discuss in detail the different EMMPRIN binding partners and elucidate their potential biological functions.

### 3.1. Caveolin-1 and Cyp60

The interaction of EMMPRIN with caveolin-1 occurs via the EC2 domain of lowly glycosylated EMMPRIN. Binding between caveolin-1 and EMMPRIN decreases MMP-1 activity. By binding to EMMPRIN, caveolin-1 inhibits the conversation of lowly glycosylated to highly glycosylated EMMPRIN, but only the highly glycosylated form stimulates MMP production [14,28]. Binding between caveolin-1 and EMMPRIN occurs in the Golgi complex, from where caveolin-1 translocates lowly glycosylated EMMPRIN to the cell surface [14].

In addition, EMMPRIN is required for the recruitment of caveolin-1 to lipid rafts under physiological as well as conditions inflammatory conditions, and thereby is associated with controlling vascular barrier function as response to several triggers, including tumor necrosis factor α (TNFα), vascular endothelial growth factor (VEGF) and interleukin (IL)-1β [35].

Cyclophilin 60 (Cyp60), a 60 kDa protein of the cyclophilin family, is another intracellular binding partner of EMMPRIN [26]. Cyp60 regulates the intracellular trafficking of EMMPRIN from the Golgi complex to the cell membrane. In this way, Cyp60 acts as a chaperone and mediates the surface expression of EMMPRIN via the secretory pathway. EMMPRIN and Cyp60 interact via the amino acid Pro^211^ within the transmembrane domain of EMMPRIN [26,36].

These findings show that caveolin-1 and Cyp60 are both involved in surface expression of EMMPRIN. As an upregulation of the EMMPRIN expression is found in cardiovascular disease, this implies an indirect involvement of these bindings partners in disease pathogenesis.

### 3.2. Monocarboxylate Transporter, CD98, and CD44

EMMPRIN is co-localized with monocarboxylate transporter (MCT)-1 and MCT4, involving the cytoplasmic domain of EMMPRIN. Notably, MCT1 and MCT4 require co-expression of EMMPRIN for proper folding, and stable membrane expression, so that EMMPRIN acts as a chaperon for the correct translocation and expression of MCT1 and MCT4 on the cell surface [21,37]. Absence of EMMPRIN leads to degradation of MCT4. However, MCTs and EMMPRIN are mutually dependent on each other, and in the absence of MCTs, EMMPRIN is not fully glycosylated and remains in the endoplasmic reticulum. MCTs and EMMPRIN are thus associated with each other and will only be properly expressed on the cell surface if both proteins are present [38]. MCT1 and MCT4 are both expressed in cardiac muscle in rats. mRNA and protein expression of MCT1 and MCT4 increase within 15 min after myocardial ischemia, and myocardial ischemia/reperfusion injury, and return to baseline after one hour. Shortly after reperfusion, an imbalance in glycolysis, lactate transport and energy production is observed, which leads to a change in the pH and ischemic stress in the heart. Interestingly, increased expression of MCT1 and MCT4 is accompanied by an elevated expression of EMMPRIN during ischemia/reperfusion [39].

Another interaction partner for EMMPRIN from the MCT family is MCT11, which is involved in lipid metabolism. Similar to MCT1 and MCT4, EMMPRIN acts as a chaperon protein for the correct translocation of MCT11 to the cell surface. A decrease in MCT11 expression is associated with an elevated risk of diabetes mellitus due to changes in lipid metabolism in human hepatocytes [40]. As patients with coronary artery disease display higher platelet-oxLDL levels compared to healthy controls [41], this may suggest that changes in lipid metabolism can impact LDL uptake by platelets and thus increase the risk for cardiovascular events.

CD98 is a type II transmembrane protein and consists of a heavy chain (CD98hc) and a light chain and associates with EMMPRIN via the EC1 domain [22,42]. Moreover, EMMPRIN seems to be involved in regulating the surface expression of CD98. Downregulation of EMMPRIN reduces the surface expression of CD98 without altering its mRNA expression. By contrast, downregulation of CD98 leads to upregulation of EMMPRIN mRNA and membrane protein expression levels [43]. A prerequisite for this interaction is a high degree of glycosylation of EMMPRIN. Besides EMMPRIN, CD98 forms complexes with MCTs and the amino acid transporter LAT1. These interactions (EMMPRIN with CD98hc, CD98hc with LAT1, and EMMPRIN with MCTs) result in the formation of a large MCT-EMMPRIN-CD98hc-LAT1 complex, which plays a critical role in cellular energy metabolism [22]. Of note, CD98hc is upregulated during plaque formation in low density lipoprotein receptor deficient (Ldlr^−/−^) mice in vivo, and the presence of CD98hc in the plaque leads to an elevated migration of vascular smooth muscle cells and thus a stabilization of the atherosclerotic plaque [42,44].

CD44 can bind EMMPRIN as well as MCT1 and MCT4 on the cell surface. Treatment with hyaluronan oligomers or a down-regulation of EMMPRIN leads to an internalization of CD44 and MCTs from the cell surface, thus affecting lactate homeostasis. However, EMMPRIN is not necessary for the cell surface expression of CD44 and loss of EMMPRIN does not affect CD44 expression [23].

Taken together, these data imply that the interactions of EMMPRIN with MCTs, CD98 and CD44 are involved in the energy metabolism of the cells, especially lactate homeostasis. A disturbance of these interactions could lead to cell damage.

### 3.3. Integrins, CD43, Annexin-2 and NOD2

The integrins α^3^β_1_ and α^6^β_1_ co-localize with EMMPRIN on the cell surface and bind to EMMPRIN as revealed by co-immunoprecipitation of cell lysates. In these interactions, EMMPRIN primarily binds to the β_1_ integrin [32]. Of note, β_1_ integrin can also interact with CD98 [45].

Another binding partner of EMMPRIN is CD43, a sialoglycoprotein, which can also interact with LFA-1 (CD11a/CD18). EMMPRIN itself is not able to bind to the CD18 subunit of LFA. Through its interaction with EMMPRIN, CD43 regulates EMMPRIN-induced cell adhesion of leukocytes [24]. After myocardial infarction in rats, an increase in CD43^+^ monocytes can be observed after 1 day with a maximum upregulation at day 3 after surgery compared to the baseline [46]. This suggests that the upregulation of CD43 mediates the EMMPRIN-dependent adhesion of leukocytes in the infarcted area after myocardial infarction.

Annexin-2 is an F-actin-binding protein and is present in the plasma membrane and endosomal vesicles. The interaction of EMMPRIN with annexin-2 on the cell surface is involved in the migration and invasion of human hepatoma cells. Knockdown of annexin-2 reduces the migratory potential of human hepatocellular carcinoma cells and MMP production in fibroblasts. By interacting with F-actin, annexin-2 could here function as a link to regulate the EMMPRIN-induced migration and infiltration of cancer cells [29,47].

NOD2 is an intracellular binding partner of EMMPRIN and is important during bacterial infections. The presence of NOD2 close to the cell surface is important for the early response after bacterial infection and the EMMPRIN-NOD2 complex seems to be involved in innate immune responses [30].

### 3.4. Cyclophilin A

Cyclophilin A (CyPA) is an 18 kDa chaperon protein expressing peptidyl-prolyl *cis*–*trans* isomerase (PPIase) activity [48]. Intracellularly, CyPA regulates protein folding, trafficking, and Ca^2+^ signaling [49,50,51,52] and has been shown to be involved in hemostasis and thrombosis, which are impaired in CyPA^−/−^ mice [53].

Besides its intracellular functions, CyPA can be released into the extracellular space from various cell types, and secreted CyPA acts as a DAMP (danger associated molecular pattern), critically regulating inflammation and thrombosis [54,55,56,57,58]. Due to the high amount of intracellular CyPA, platelets are a major source of extracellular CyPA during inflammation [55,59]. But CyPA can also be released by other cell types upon inflammatory stimulation, e.g., vascular endothelial cells, lipopolysaccharides (LPS)-activated macrophages, or monocytes exposed to high glucose concentrations [54].

**Figure 1:**
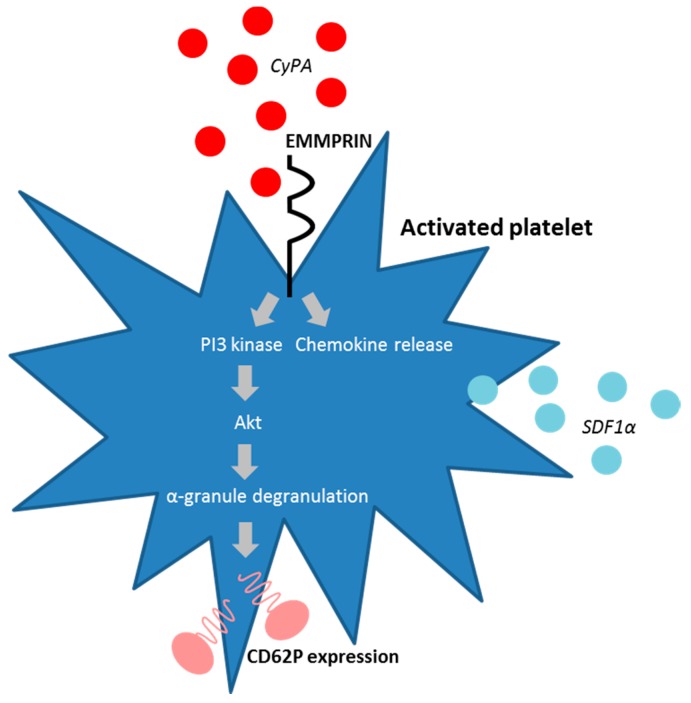
CyPA is able to activate platelets by binding to EMMPRIN. CyPA binding to EMMPRIN activates the PI3 kinase/Akt pathway and induces α-granule degranulation and thus surface expression of CD62P. Additionally, the interaction of CyPA and EMMPRIN induces the expression of the chemokine SDF1α.

**Figure 2:**
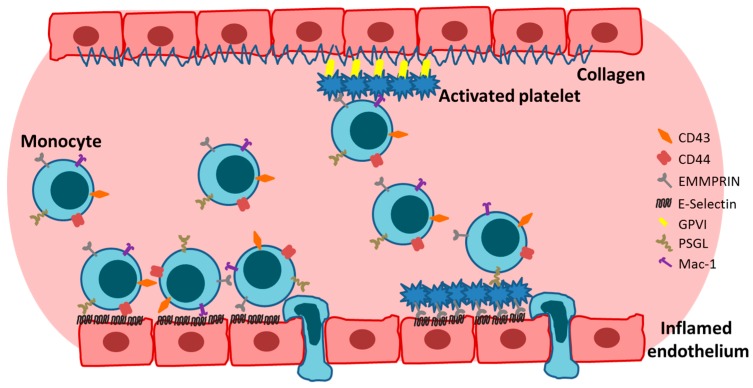
Monocytes and platelets are able to interact with inflamed endothelium and collagen via several receptors. Moreover, endothelial-bound platelets are able to capture monocytes. Adherent monocytes can then migrate into the tissue.

**Figure 3:**
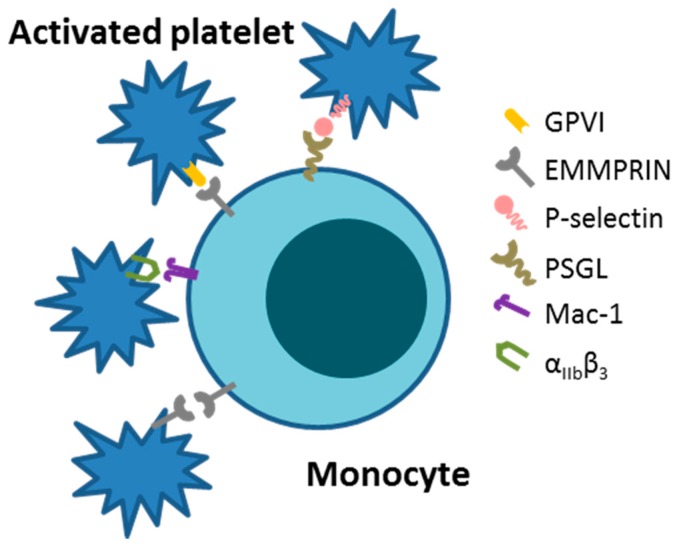
Monocytes and platelets can bind to each other via several receptors. The main interaction is via P-selectin and PSGL. In addition, EMMPRIN is able to form monocyte platelet aggregates (MPAs) by interacting with other EMMPRIN receptors or GPVI. Platelet α_IIb_β_3_ is able to bind to Mac-1 on monocytes.

**Figure 4:**
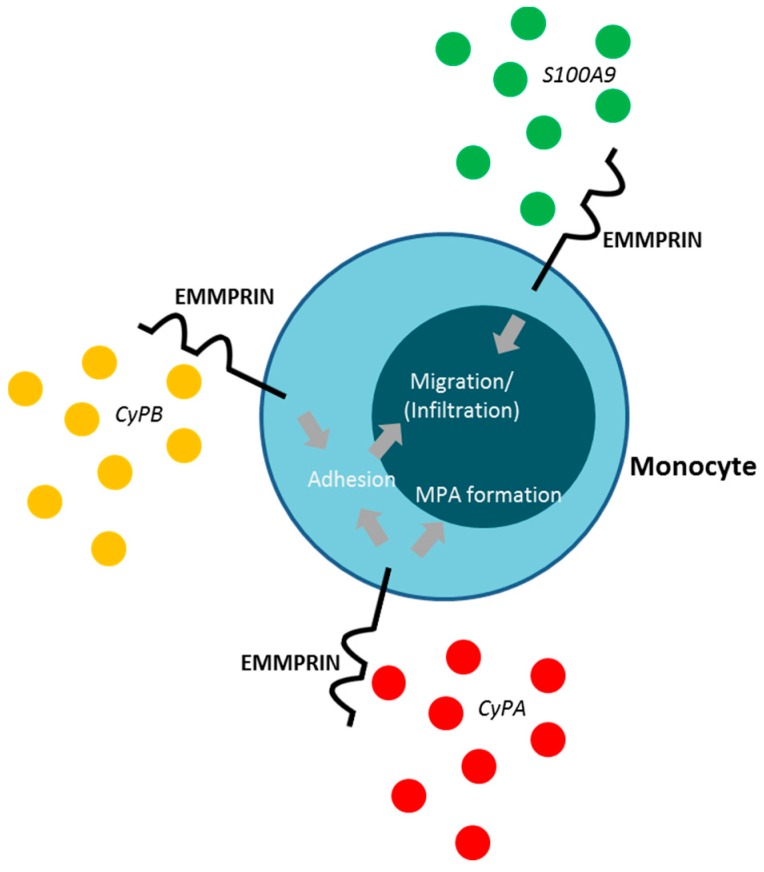
The interaction of the extracellular ligands CyPA, CyPB and S100A9 induce the adhesion and migration/infiltration of monocytes. Moreover, binding of CyPA to EMMPRIN induces the formation of MPA.

First described in 2002, extracellular CyPA acts as a specific ligand for the receptor EMMPRIN [18]. Binding of CyPA to EMMPRIN is independent of CyPA PPiase activity [60]. After activation, platelets express EMMPRIN on their cell surface within a few minutes in vitro, and an increase in EMMPRIN expression can also be found on the cell surface of blood monocytes after acute myocardial infarction [16,61]. Activated platelets display elevated levels of surface-bound CyPA [62], possibly due to binding of extracellular CyPA to EMMPRIN. CyPA binding to EMMPRIN induces platelet degranulation and activation via the PI3 kinase/Akt pathway (Figure 1). This activation leads to a shape change of platelets and the release of pro-inflammatory chemokines like stromal cell-derived factor (SDF) 1α into the extracellular space. Moreover, CyPA-activated platelets aggregate at the injured vessel wall in vivo (Figure 2) [59]. CyPA-activated platelets furthermore bind to monocytes and form platelet-monocyte aggregates (MPAs) [63], possibly by inducing P-selectin expression on platelets mediated by CyPA-EMMPRIN interactions [59], interacting with P-selectin glycoprotein ligand-1 (PSGL-1) on monocytes. These interactions may facilitate the first contact of platelets and leukocytes, whereas further interactions are mediated by other receptors expressed on platelets and leukocytes, including the receptors Mac-1 (α_M_β_2_, CD11b/CD18) and α_IIb_β_3_ on platelets (Figure 3) [64,65,66]. Activated platelets bind to inflamed endothelium and can furthermore capture circulating leukocytes, leading to endothelium-platelet-monocyte interactions (Figure 2) [67]. MPA formation triggers the release of several inflammatory cytokines and chemokines, including CCL2 and IL-8 [68]. CyPA also plays a role in monocyte activation via EMMPRIN, entailing an increase in CD11b surface expression [63]. The interaction of CyPA with EMMPRIN is also involved in chemotaxis of neutrophils, monocytes, eosinophils and T-cells (Figure 4) [56,69,70] and can be impeded by the inhibition of EMMPRIN or disturbance of the CyPA-EMMPRIN interaction in vitro and in vivo. Based on these findings and the fact that CyPA induces thrombus formation in vitro and in a FeCl_3_ model in vivo, it can be speculated that the CyPA-induced activation of platelets via EMMPRIN is an essential step in inflammation and the development of atherosclerosis [63] and other cardiovascular diseases.

Indeed, an increased EMMPRIN and CyPA expression is observed in atherosclerotic plaques in mice fed a high-fat diet [71], and double knockout mice deficient in apolipoprotein E (ApoE) and CyPA are protected against the development of atherosclerosis. CyPA promotes the development of atherosclerotic lesions, possible via its expression by monocytes/macrophages in response to inflammation, and its chemoattractant activities, leading to an accelerated infiltration of monocytes into the atherosclerotic plaque [18,72,73]. In human atherosclerotic plaques, EMMPRIN is expressed in macrophage-rich areas [11], suggesting an elevated expression of EMMPRIN in macrophages. The expression of EMMPRIN and CyPA increase during the differentiation of monocytes into macrophages and foam cells [11,71]. The interaction of EMMPRIN and CyPA has furthermore been reported to increases MMP-9 activity [71] via activation of NF-κB [74]. Upregulation of MMP-9 expression is prominent in macrophages compared to monocytes, in line with the increase in EMMPRIN and CyPA expression during differentiation [75]. Notably, significantly higher EMMPRIN expression levels were reported on platelets from patients with stable and unstable coronary artery disease (CAD) compared to healthy controls, so that a direct correlation of platelet EMMPRIN expression and CAD has been proposed [76].

Patients with acute myocardial infarction display an increased monocyte surface expression of EMMPRIN [77], also on different monocyte subsets [61], as well as enhanced plasma MMP-9 levels [77]. Moreover, an elevated expression of EMMPRIN can be found on cardiomyocytes and CyPA in infiltrating leukocytes in the infarcted area in patients after myocardial infarction. Inhibition of EMMPRIN by a neutralizing antibody and deficiency in CyPA in mice protects against ischemia and reperfusion injuries [78], suggesting an important role of the interaction of EMMPRIN and CyPA during ischemia/reperfusion injury. NAD(P)H oxidase subunit p47^phox^-derived reactive oxygen species (ROS) are involved in ischemia/reperfusion damage, and blocking of EMMPRIN reduces oxidative stress after myocardial infarction in vivo [79]. Besides this, angiotensin II-induced ROS generation is impaired in mice deficient in ApoE and CyPA, implying a regulatory role of CyPA in ROS generation by interacting with p47^phox^ [72,80].

In addition to its role in cardiovascular diseases, the interaction of CyPA and EMMPRIN is also involved in the development and progression of other inflammatory diseases. The presence of extracellular CyPA contributes to inflammatory processes by promoting the secretion of pro-inflammatory cytokines such as TNF-α, IL-1β, IL-8 and CCL2 from monocytic cell lines [74]. Due to these inflammatory properties, CyPA is involved in various diseases including rheumatoid arthritis [74] and sepsis [81], where platelets also play a role in disease progression [82]. In rheumatoid arthritis, EMMPRIN is involved in joint destruction due to an enhanced expression of MMPs [83], induced by the interaction of CyPA and EMMPRIN. Moreover, blocking of EMMPRIN in a mouse sepsis model reduces sepsis-induced renal failure by preventing binding of CyPA to its receptor [84]. Based on the inflammatory properties of CyPA and the presence of CD68^+^ cell infiltrates in cardiac biopsies of patients with tachycardia-induced cardiomyophathy (TCM) compared to healthy heart tissue, it can be speculated that an increased expression of CyPA may also be found in human TCM [85].

Interestingly, CyPA can be methylated, acetylated or phosphorylated [86]. Endogenous CyPA is acetylated in Lysine^125^ in human cell lines, HeLa and Jurkat T cells. Between one third and half of the endogenous CyPA is acetylated depending on the cell line. Acetylated CyPA has a different optimal pH range than unacetylated CyPA, whereas CyPA needs a basic environment, acetylated CyPA requires an acidic environment. Acetylation does not lead to a confirmation change of CyPA. However, acetylated CyPA has a lower binding affinity for cyclosporine A, inhibits calcineurin and interferes with the HIV-1 capsid interaction compared to unacetylated CyPA [87]. Moreover, there is upregulation of acetylated CyPA during hypoxia, where it interacts with ATG5 and ATG7, key promotors for autophagy. Autophagic processes in turn lead to abnormal angiogenesis and promote the progression of hypoxic pulmonary arterial hypertension [88]. Acetylated CyPA can also be secreted into the extracellular space from endothelial cells in response to oxidative stress induced by angiotensin II and enhances endothelial inflammation. Extracellularly, acetylated CyPA is superior to unacetylated CyPA in inducing pro-inflammatory signal transduction pathways and apoptosis of endothelial cells. The reason for this finding could be that acetylated CyPA leads to a change in the electrostatic surface. However, both acetylated CyPA and unacetylated CyPA are involved in the development of pulmonary arterial hypertension [89,90]. Besides its acetylation, CyPA can also be phosphorylated in response to ligand stimulation via CXCR4. In addition, CyPA is involved in the internalization of CXCR4 after SDF1α treatment. The intracellular interactions of CyPA and CXCR4 lead to the translocation of CyPA to the nucleus where CyPA binds to the heterogeneous nuclear ribonucleoprotein (hnRNP) A2. This interaction is involved in the nuclear export of hnRNP A2, the nuclear translocation of the Erk1/2, and thus the migration of cells towards SDF1α [91].

### 3.5. Apolipoprotein D

EMMPRIN is involved in the internalization of apolipoprotein D (ApoD) in vitro and a reduction of EMMPRIN downregulates this internalization. ApoD interaction with EMMPRIN requires EMMPRIN to be lowly glycosylated [27]. ApoD is reported to have a protective role in the response to stressful stimuli, like oxidative stress, especially lipid peroxidation, and its expression is upregulated in atherosclerotic plaques in ApoE^−/−^ mice, Alzheimer disease and cancer [92,93,94]. However, CyPA and ApoD compete for binding to EMMPRIN, because increased extracellular CyPA levels reduce the internalization of ApoD and EMMPRIN in the cell. This indicates that binding of ApoD and EMMPRIN reduces CyPA-mediated inflammatory responses [27]. In atherosclerotic plaque tissue, downregulation of ApoD gene expression can be observed, which may indicate reduced protective effects by ApoD and unchecked CyPA-mediated proinflammatory effects in atherosclerosis [95].

### 3.6. Cyclophilin B and Syndecan-1

Like CyPA, CyPB is ubiquitously expressed as an intracellular protein but shows lower expression levels than CyPA. CyPB displays PPiase activity, which is involved in binding of CyPB to EMMPRIN [96,97,98,99]. Intracellular CyPB is mainly located in the endoplasmic reticulum [98] and is secreted into the extracellular space upon activation [19].

CyPB has pro-inflammatory as well as anti-inflammatory effects. CyPB-EMMPRIN interactions activate the Erk pathway and protect against oxidative stress [99]. However, CyPB also mediates the adhesion and chemotaxis of T-cells, neutrophils, monocytes and platelets via EMMPRIN in vitro (Figure 4) [19,69,100]. However, CyPB is not involved in platelet aggregation or degranulation [100]. Moreover, CyPB cannot induce secretion of pro-inflammatory cytokines with the exception of IL-6. The expression of IL-6 suggests that CyPB acts as a pro-inflammatory mediator by recruiting inflammatory cells. In contrast, pre-treatment of macrophages with CyPB significantly reduces the LPS-induced expression of pro-inflammatory cytokines, like TNFα, IL-8 and IL-1β, but does not affect the production of IL-6 and IL-10. The inhibitory effect of CyPB on TNFα expression is dependent on a transcriptional mechanism, which involves expression of B-cell lymphoma 3 [101].

On T-cells, it could be shown that syndecan-1 is associated with EMMPRIN and that this interaction is stabilized in the presence of CyPB. An inhibition of syndecan-1 reduces the CyPB-induced migration of T-cells and THP-1 cells and prevents the CyPB-mediated activation of the Erk pathway. This indicates that interaction of syndecan-1 and EMMPRIN is necessary for the pro-inflammatory effects of CyPB [31].

### 3.7. S100A8/S100A9 Complex

S100A9 is intracellularly located in the cytosol [102] and belongs to the S100 family. It is an EF-hand Ca^2+^-binding protein [103,104]. S100A9 is able to form a heterodimer with S100A8, which is also the preferred heterodimerization partner in human as well as in mice [105].

S100A9 mediates the adhesion and migration/infiltration of monocytes and neutrophils in vitro and in vivo (Figure 4) within a few hours, which suggests pro-inflammatory properties [106]. The pro-inflammatory properties of S100A9 are supported by the fact that there is co-localization of S100A9 with macrophages/granulocytes in human thrombi and that there is an increase in S100A9 mRNA expression in human platelets in patients with acute coronary syndrome (ACS). In addition, S100A9 serum levels are markedly elevated in ACS patients [107,108]. However, S100A9 is not secreted by cardiac myocytes during hypoxia and is thus independent of the ischemic event itself. This suggests that the origin of the extracellular/membrane bound S100A9 likely is secreted S100A9 released from infiltrated cells [109].

The interaction between S100A9 and its receptors activates pro-inflammatory signal cascades and promotes the development and progression of several diseases, including rheumatoid arthritis [104,110]. However, most effects of S100A9 were investigated by studying the interaction of S100A9 with the receptors Toll-like receptor (TLR)-4 or RAGE [111,112] as binding of S100A9 to EMMPRIN was only recently discovered in 2012 [20]. The interaction of S100A9 with EMMPRIN induces the migration of monocytes and possibly also cancer cells via the EMMPRIN-Erk pathway [20,113]. Currently, it is still under debate whether the interaction of S100A9 and EMMPRIN induces pro-inflammatory cytokine expression. In cancer cells, this interaction induces the secretion of pro-inflammatory factors, including TNF-α, IL-8 and MMP-1 [20]. In contrast, the secretion of pro-inflammatory cytokines in monocytes is independent of the S100A9 EMMPRIN interaction [113]. However, the investigated cytokines differ in these two studies, so it cannot be excluded that TNF-α, IL-8 and MMP-1 would also be expressed by S100A9-treated monocyte via EMMPRIN.

In the S100A8/A9 complex, S100A9 is the important player for thrombosis. It is secreted into the extracellular space and binds to several receptors including EMMPRIN, RAGE, TLR4 and CD36. Secreted S100A9 binds to platelets after thrombin stimulation and is responsible for its pro-thrombotic actions in vitro and in vivo. The S100A9-induced effects on platelets are CD36 associated. However, these effects have not been investigated with regards to the involvement of EMMPRIN [20,114].

Besides these pro-inflammatory properties of S100A9, there are also anti-microbial effects associated with S100A9 expression, as in pneumonia-induced sepsis, where S100A9 reduces bacterial growth [115]; whether this effect is mediated by EMMPRIN is similarly still unknown.

S100A9 is the ligand of several receptors and there are only a few studies which show the direct involvement of EMMPRIN in cytokine release and thrombosis. However, the interaction of S100A9 with EMMPRIN mediates the migration of monocytes and the secretion of pro-inflammatory cytokines.

### 3.8. Glycoprotein VI

Glycoprotein VI (GPVI) was first described on platelets in 1982 [116] and was discovered as a binding partner for EMMPRIN by Seizer et al. in 2009 [10]. GPVI is upregulated on the platelet surface in acute coronary syndromes [117] and on monocytes after acute myocardial infarction [77]. The GPVI-EMMPRIN interaction mediates the rolling of cells and thus the first step in binding between cells during monocyte extravasation. Based on these findings, Seizer et al. [10] speculated that platelet GPVI can form monocyte-platelet aggregates (MPA) by binding to EMMPRIN on monocytes [10]. Indeed, an interaction between platelet GPVI and monocyte EMMPRIN and thus the formation of MPA could subsequently be demonstrated in 2011 [34]. Besides binding to monocytes in MPA (Figure 3), platelets can also form complexes with other immune cells, including neutrophils [118]. The interaction of platelets with monocytes occurs within the circulation [119] and the formation of MPA is increased after cardiovascular events. For instance, more circulating MPA can be found in patients after acute myocardial infarction compared to patients without infarction or healthy controls [120,121]. However, even weak inflammatory stimuli, like influenza immunization, leads to an increase in MPA formation within the circulation. Monocytes in humans can be classified in three groups, i.e., CD14^++^CD16^−^, CD14^++^CD16^+^ and CD14^−^CD16^++^ subsets, and all monocyte subsets are able to interact with platelets. However, the interaction of platelets with monocytes leads to a change in their phenotype, and monocytes convert from CD14^++^CD16^−^ to the CD14^++^CD16^+^ phenotype, with CD14^++^CD16^+^ monocytes displaying an increased expression of CD11b and CD11c, indicative of monocyte activation. In addition, CD14^++^CD16^+^ monocytes as well as CD14^++^CD16^+^ MPA adhere better to TNFα-activated endothelium compared to the other monocyte subsets [122,123]. Patients with stable CAD and acute myocardial infarction show an increased number of CD16^+^ monocytes [61], and these patients also show significantly elevated levels of MPA formation among all monocyte subsets. In particular, CD16^+^ monocytes have a higher potential to form MPA [123]. Beside CAD [123,124], there is also an increase in MPA formation in other cardiovascular diseases like acute myocardial infarction [125] and ischemic heart failure [126], and MPA formation is associated with risk factors for cardiovascular diseases, such as hypertension [127] and smoking [128]. In acute myocardial infarction, elevated EMMPRIN levels are found on all monocyte subsets compared to stable CAD [61], which suggests that the increase of MPA is associated with an increase in EMMPRIN levels in monocytes and thus enhanced binding of platelet GPVI to monocyte EMMPRIN.

Moreover, EMMPRIN expression on monocytes mediates the adhesion of monocytes to adherent platelets under venous and arterial shear flow conditions, thereby promoting the platelet-induced adhesion of monocytes to the vascular wall (Figure 2) [34]. Furthermore, clinical data revealed a correlation of soluble GPVI with soluble EMMPRIN in the plasma of healthy controls and patients with cardiovascular diseases [129].

### 3.9. E-Selectin

E-selectin is expressed by endothelial cells after stimulation in a dose- and time-dependent manner. The maximum RNA expression of E-selectin can be observed three to four hours after stimulation and levels then decrease to baseline levels within 24 h [130].

E-selectin promotes the rolling of platelets on inflamed endothelium in vivo. This interaction of platelets with the inflamed endothelium is independent of the presence of platelet P-selectin. This implies that there is another receptor on platelets that is able to induce rolling/binding of platelets to the endothelium [67]. EMMPRIN expressed on platelets can serve as a binding partner of E-selectin [16,25] giving rise to the possibility that the rolling of platelets is induced by the interaction of platelet EMMPRIN with endothelial E-selectin (Figure 2).

In addition, E-selectin and attached platelets on the endothelium promote the rolling of neutrophils over stimulated endothelial cells [67,131] and thus their infiltration into inflamed areas. By this mechanism, EMMPRIN on neutrophils and endothelial E-selectin also promote the infiltration of neutrophils into an infarcted area. This could be shown in renal ischemia/reperfusion experiments, where the infiltration of neutrophils is trigger by an increased expression of the adhesion molecule E-selectin in capillary blood vessels in the infarcted area of the kidney [25]. It could furthermore be shown that there is an increase in E-selectin expression after myocardial infarction in vivo [132], and in the presence of E-selectin, an increased neutrophil infiltration in the infarcted area could be found, suggesting a role of E-selectin in binding of neutrophils to the activated/damaged endothelium and thereby the development of ischemia/reperfusion injuries [132]. The interaction of EMMPRIN and E-selectin could also be involved in ischemia/reperfusion injuries after myocardial infarction. Blocking of EMMPRIN leads to a decrease in ischemia/reperfusion injury and reduced accumulation of infiltrated monocytes and macrophages in the infarcted area [78]. This suggests that similar to renal ischemia/reperfusion injury, an increase in E-selectin expression in the myocardium triggers the infiltration of neutrophils into the infarcted area, which subsequently promotes pro-inflammatory effects, such as cell necrosis and the production of reactive oxygen species (ROS) [25,133].

### 3.10. EMMPRIN

EMMPRIN can dimerize on the same cell, which was first shown in 1996 by Fadool and Linser [134] in different chicken tissues. Later it was shown that this dimerization occurs in a *cis*-dependent manner [33], possibly mediated by strand swapping of the EC1 domain [135]. For dimer formation, EMMPRIN has to be highly glycosylated [14]. Egawa et al. [17] showed that MT1-MMP can cleave a 22 kDa fragment of the extracellular EC1 domain from the cell surface. Also, MT2-MMP can cleave EMMPRIN but not as effectively as MT1-MMP [17]. Additionally, Ellis et al. [5] found two different forms of EMMPRIN in the supernatant of cells, a 35 kDa cleaved fragment and the secreted 58 kDa form. Cleavage of the dimerization site can lead to a down-regulation of the cellular functions of EMMPRIN due to a lack of EMMPRIN-EMMPRIN interactions [17]. However, EMMPRIN can still activate intracellular pathways by forming dimers with other EMMPRIN receptors [16]. Soluble EMMPRIN is able to bind to surface bound EMMPRIN on cells via the EC1 domain. Due to this interaction, other EMMPRIN binding partners, which also bind to the EC1 domain, are blocked from this interaction. These results in a decrease in cytokine release at least in vitro compared to agonist binding of EMMPRIN [20]. Moreover, soluble EMMPRIN can be internalized by binding to surface-bound EMMPRIN receptors. This mechanism is dependent on the presence of surface-bound EMMPRIN. The binding between soluble and surface-bound EMMPRIN increases the surface expression of EMMPRIN and upregulates its endogenous levels. However, the soluble form of EMMPRIN is not able to form dimers [15].

Biswas et al. showed that the co-culture of tumor cells with fibroblasts led to an increase in type I collagen-degrading enzyme secreted from fibroblasts [2]. This indicates that there is a cell–cell interaction between tumor cells and fibroblasts. EMMRPIN as a receptor responsible for the collagen-degrading activity of tumor cells was already identified in 1989 [5]. The counter-receptor on fibroblasts, however, was still unknown. EMMRPIN-stimulated fibroblasts produce MMP-1, MMP-2, MMP-3 and MT1-MMP in melanoma-fibroblast co-culture experiments and inhibition of EMMPRIN reduces the MMP expression from fibroblasts. In addition, the fact that there is a correlation between the EMMPRIN expression on melanoma cells and the expression of MMPs by fibroblasts indicates the existence of a counter-receptor for EMMPRIN on fibroblasts [136]. EMMPRIN as its own counter-receptor was first described in 2001. That EMMPRIN is a binding partner for itself was also shown in dynamic adhesion experiments in which EMMPRIN-transfected CHO cells were perfused over EMMPRIN-Fc. In a second experiment, platelets were blocked with an anti-EMMPRIN antibody and then perfused over EMMPRIN-Fc. Both experiments show that EMMPRIN can bind to other EMMPRIN receptors [10]. For this hemophilic cell-cell interaction, the EC1 domain is necessary as revealed by use of different EMMPRIN inhibitors. Inhibition of the hemophilic interaction also shows a reduction in MMP production and MMP-dependent tumor cell invasion. This suggests that the hemophilic interaction of EMMPRIN is involved in the MMP production of fibroblasts [137]. However, the ability to induce MMP expression by EMMPRIN is dependent on its glycosylation state. Only the highly glycosylated form can induce MMP expression. In addition, deglycosylation of EMMPRIN prevents the induction of MMPs in vitro [28,137]. This suggests that dependent on the glycosylation state, EMMPRIN either acts as an agonist or antagonist against itself.

Treatment with angiotensin II induces the expression of MMP-9 from vascular smooth muscle cells (VSMCs) via angiotensin receptor subtype-1 (AT1) and NF-κB. The expression occurs in a time-dependent manner, and the MMP-9 expression remains elevated for more than 24 h with highest expression after 12 h [138]. Besides MMP-9, angiotensin II also increases MMP-2 expression in smooth muscles cells (SMC). However, the MMP-2 expression is oxidative stress-sensitive, and knockout of the NAD(P)H-oxidase subunit p47^phox^ inhibits MMP-2 expression completely. In left anterior descending coronary artery samples from patients with ischemic cardiomyopathy, co-localization of MMP-2, angiotensin II, p47^phox^ and AT1 could be detected [139]. Oxidative stress is involved in the expression of MMP-2, which is also relevant for myocardial infarction in mice. After a myocardial infarction, superoxide production is upregulated and there is more oxidative stress. In p47^phox^ knockout mice, no increase in superoxide production could be found and a significant improvement in the left ventricular ejection fraction was noted compared to wild type mice. Additionally, elevated MMP-2 levels were detected in the left ventricular myocardium of wild type mice during myocardial infarction as well as in atherosclerotic plaques, which suggests that MMP-2 expression is dependent on p47^phox^ [79]. This indicates that the NAD(P)H-oxidase subunit p47^phox^ is involved in the induction of MMP-2 expression in atherosclerosis and during myocardial infarction. Moreover, an increase in oxidative stress and the production of ROS leads to secretion of CyPA [140] and consequently to the binding of extracellular CyPA to EMMPRIN. The binding between CyPA and EMMPRIN and the hemophilic interaction of EMMPRIN induce the expression of MMP-9 in foam cells [71].

## 4. Conclusions

In conclusion, EMMPRIN is an important receptor during inflammation. Due to its various binding partners and functions, EMMPRIN plays a role in the development and progression of cardiovascular diseases, including atherosclerosis and myocardial infarction. In particular, the interaction with its binding partners, especially with CyPA, induces the expression of pro-inflammatory chemokines and the activation of platelets. CyPA-activated platelets are able to bind monocytes and induce monocyte-platelet aggregates in part mediated by EMMPRIN binding to GPVI. In addition, the ability to induce MMP expression strongly suggests a role of EMMPRIN in atherosclerosis and myocardial infarction. Full understanding of EMMPRIN interactions and signaling pathways in the different cell types may open up novel therapeutic approaches for treating cardiovascular diseases.

## Abbreviations

Apo	Apolipoprotein
CAD	Cardiovascular disease
CD	Cluster of differentiation
Cyp	Cyclophilin
DAMP	Danger associated molecular pattern
EMMPRIN	Extracellular Matrix Metalloproteinase Inducer
IL	Interleukin
LDLR	Low density lipoprotein receptor
LPS	Lipopolysaccharide
MCT	Monocarboxylate transporter
MMP	Matrix metalloproteinase
ROS	Reactive oxygen species
TNFα	Tumor necrosis factor alpha
